# Danger Signs of Childhood Pneumonia: Caregiver Awareness and Care Seeking Behavior in a Developing Country

**DOI:** 10.1155/2015/167261

**Published:** 2015-10-21

**Authors:** Ikenna K. Ndu, Uchenna Ekwochi, Chidiebere D. I. Osuorah, Kenechi S. Onah, Ejike Obuoha, Odutola I. Odetunde, Ikenna Nwokoye, Nnenne I. Obumneme-Anyim, Ifeyinwa B. Okeke, Ogechukwu F. Amadi

**Affiliations:** ^1^Department of Paediatrics, Enugu State University of Science and Technology, Enugu State, Nigeria; ^2^Child Survival Unit, Medical Research Council UK, The Gambia Unit, Fajara, Gambia; ^3^Department of Paediatrics, Nnamdi Azikiwe University Teaching Hospital, Anambra, Nigeria; ^4^Department of Paediatrics, University of Nigeria Teaching Hospital, Enugu State, Nigeria; ^5^Department of Paediatrics, Federal Teaching Hospital Abakaliki, Ebonyi, Nigeria

## Abstract

*Background.* Efforts to reduce child mortality especially in Africa must as a necessity aim to decrease mortality due to pneumonia. To achieve this, preventive strategies such as expanding vaccination coverage are key. However once a child develops pneumonia prompt treatment which is essential to survival is dependent on mothers and caregiver recognition of the symptoms and danger signs of pneumonia.* Methods.* This community based cross-sectional study enrolled four hundred and sixty-six caregivers in Enugu state. It aimed to determine knowledge of caregivers about danger signs of pneumonia and the sociodemographic factors that influence knowledge and care seeking behaviour of caregivers.* Results.* There is poor knowledge of the aetiology and danger signs of pneumonia among caregivers. Higher maternal educational attainment and residence in semiurban area were significantly associated with knowledge of aetiology, danger signs, and vaccination of their children against pneumonia. Fast breathing and difficulty in breathing were the commonest known and experienced WHO recognized danger signs while fever was the commonest perceived danger sign among caregivers.* Conclusion.* Knowledge of danger signs and health seeking behaviour among caregivers is inadequate. There is need for intensified public and hospital based interventions targeted at mothers to improve their knowledge about pneumonia.

## 1. Introduction

Pneumonia causes almost 1 in 5 under-five deaths worldwide and is responsible for more than 2 million deaths of children each year [1]. Nigeria, with a predicated incidence of 6.1 million pneumonia cases and 0.38 episodes of pneumonia per child-year in 2008, ranks 5th among the 15 countries with highest pneumonia burden in the world [2]. It is a leading cause of childhood morbidity and mortality in developing countries including Nigeria [3]. In 2010, pneumonia accounted for 868,000 deaths in under-5 children, which was 14% of all causes of deaths in children [4]. However when detected early, pneumonia can be treated with easily available medication at a low cost [5]. Unfortunately, only one in five caregivers in developing countries can recognize the danger signs of pneumonia like rapid breathing and chest indrawing, and only half of children under 5 with pneumonia are taken to an appropriate healthcare provider [6]. In a population based survey carried out in six countries in Sub-Saharan Africa, only about 18% and 30% of children with suspected pneumonia in Nigeria and Ethiopia, respectively, were taken to hospital for healthcare [7]. In a systematic review of ninety-one studies that reported recognition and/or care seeking for diarrhoea, pneumonia, and malaria in low and middle income countries (LMICs), it was shown that the median sensitivity of recognition of pneumonia among caregivers was low (45.8%) and care seeking from community health worker for pneumonia was 4.2% [8]. The World Health Organization (WHO) and UNICEF have recommended strengthening family's capacity to recognize danger signs and prompt care seeking as one of the interventions for controlling pneumonia in children under five [9]. This study seeks to determine the maternal perception and care seeking behaviour concerning danger signs of childhood pneumonia. It is hoped that the findings of this study will help reduce mortality from pneumonia in children and will contribute to achieving the sustainable development goal on child mortality.

## 2. Methodology

### 2.1. Study Area

This study was conducted in Enugu state, the host town of Enugu State University of Science and Technology. It is located in South East Nigeria on latitude 6° 27N and longitude 7° 30E [10]. The economy of Enugu state is dependent mainly on national oil revenue and commerce. Enugu state is made up of 17 local government areas with its capital carved from Enugu North, Enugu South, and Enugu East LGAs. The majority of the inhabitants are of Igbo ethnicity, and Christianity is the dominant religion. The minimum monthly income is similar to the national average of ₦18,000 (90 US dollars; National Minimum Wage Act 2011). Literacy rate is 66%, which is higher than the national literacy rate of 45%, and there are 955 males per 1,000 females [11].

### 2.2. Study Subject and Sampling Technique

This was a community based cross-sectional descriptive and analytical study. The study was conducted over a 10-month period in 4 of the 17 local government areas (LGAs) of Enugu state. Multistage sampling method was used to select study participants. The LGAs were divided into rural and semiurban categories based on level of development and population in the LGAs. Simple random sampling using balloting method was used to select 2 LGAs from each category.

In the second stage, one community was selected from each LGA using simple random sampling (i.e., one community from each of the two rural and two semiurban LGAs). In each of the communities visited, through adequate mobilization using the community leaders, the women were asked to gather at a convenient square. Women who were caring and/or had cared for a child in the past 2 years and consented to participate were consecutively enrolled. This process was repeated for each of the four communities visited. Three hundred and nineteen (319) caregivers who met the inclusion criteria and gave consent to participate were interviewed in the rural communities while one hundred and forty-seven (147) were interviewed in the semiurban communities.

## 3. Measures

### 3.1. Sociodemographic Characteristics

The following sociodemographic variables of respondents were assessed: (i) age grouped into 20–30, 31–40, and >40 years, (ii) highest educational attainment grouped into tertiary, secondary, primary, and no education, (iii) number of living children grouped into none, 1–4, and >4 children, and (iv) socioeconomic class: defined as the wealth index of the household derived using maternal and paternal highest educational attainment and occupation based on Oyedeji classification [12].

### 3.2. Knowledge of Pneumonia and Health Seeking Behaviour of Respondents

Pneumonia is inflammation of one or both lungs caused by variety of organisms and chemical substances characterized by cough, shortness of breath, fever and difficult breathing, cyanosis, and death in severe cases. Pneumonia is also called “oyi” in the local Igbo language and this name was used for respondents that do not understand English. The World Health Organization (WHO) recognizes four features as danger signs in pneumonia. They include stridor, fast breathing, chest wall indrawing, and difficulty in breathing (labored breathing). Knowledge of danger signs of pneumonia was assessed using open ended questions based on WHO definition [9]. Based on their responses, the respondents were grouped into no knowledge of danger signs and knowledge of at least one danger sign. Also knowledge and uptake of vaccine against pneumonia were assessed. The respondents were grouped into “Yes” for those who were aware of the danger signs and had vaccinated their children and “No” for those who had not heard about pneumonia and had not vaccinated their children. Health seeking behavior of respondents was assessed to determine where care was sought for a child with suspected pneumonia and the treatment given.

### 3.3. Data Cleaning and Analysis

Quality control check to detect errors in questionnaire administration and data recording was done by researchers on daily basis after enrollment. Where there are errors detected, the interviewers were asked to clarify them accordingly with the interviewed mother or caregiver. Microsoft excel 2007 was used to input the raw data. Data cleaning to remove grossly incomplete and/or inconsistent data was also done by researcher assistants and the study researchers. SPSS version 20 was used for data analysis. Chi-square and Fisher exact test where appropriate were used in data analysis. Results were presented as percentages and 95% confidence intervals where appropriate. Statistical significance was set at *P* value < 0.05.

### 3.4. Ethical Consideration

Ethical clearance was obtained from the Enugu State University Teaching Hospital Ethics Committee. Informed consent (written) was obtained from every mother in her own right and on behalf of her child before recruitment. Participation in the study was entirely voluntary and no financial inducement whatsoever was involved. All information was handled with strict confidentiality.

## 4. Result

### 4.1. Characteristics of Study Participants

Four hundred and sixty-six were found eligible and successfully interviewed. Most of the respondents 166 (37.4%) were 40 years and above while those in the 20–30 and 31–40 years of age bracket were 158 (35.6%) and 120 (27.0%), respectively. Approximately half of the respondents 278 (49.7%) had primary school or no formal education and 280 (60.1%) in the lower socioeconomic class. Respondents in the middle and upper socioeconomic class made up 103 (22.1%) and 83 (17.8%) of respondents. Almost two-fifth, 166 (39.0%) of the respondents, have more than 4 children and 246 (57.7%) with 1–4 children. Fourteen (3.3%) of the respondents had no children at all but had cared for one or more children in the past. In all, 211 children 5 years or younger of respondents with a mean age of 13.7 months were involved in the study (Table 1).

### 4.2. Knowledge of Pneumonia

About 95% of the respondents (440/464) had heard of pneumonia and the remaining 24 (5.2%) never heard about it. When asked about the cause, only 18 (4.1%) correctly stated its etiology. Majority of the respondents 394 (88.7%) believe pneumonia is caused by exposure to cold environment and/or ingestion of cold fluid or food while 32 (6.9%) have no idea what the cause of pneumonia is. The correlation (*r*) between awareness of pneumonia and knowledge of its cause is 0.374 (*P* = 0.001). For respondents who proffered an answer whether correct or incorrect, source of information was via electronic media (i.e., radio and/or television) in 123 (26.5%), through the Internet in 6 (1.3%), and during medical counseling in 226 (48.7%) respondents. Other sources of information include print media in 13 (2.8%), social interactions in 73 (15.7%), schools in 37 (8.0%), churches in 8 (1.7%), and others in 18 (3.9%) respondents. Almost all 426 (97.7%) of the 440 respondents who have heard of pneumonia acknowledged that it is a potentially dangerous illness that could lead to serious complications.

Inquiry into preventive measures against pneumonia showed that the vast majority believe that adequate clothing 127 (49.6%) and avoiding cold food and environment 38 (14.8%) were the best strategy to prevent pneumonia. Other preventive measures listed by respondents include cleanliness 24 (9.4%), feeding well 14 (5.5%), drugs 11 (4.3%), vaccination 27 (10.5%), prayer 2 (0.8%), educating caregivers 3 (1.2%), and avoiding crowded areas 3 (1.2%). Ten (3.9%) respondents did not know any preventive measure against pneumonia.

Knowledge of vaccine against pneumonia was alleged by 218 (46.8%) of respondents out of which 185 (39.7%) claimed they took the pneumococcal vaccine for their index child. Some of the reasons for nonvaccination for the 281 (60.3%) respondents who did not vaccinate their child included lack of knowledge of the availability of pneumococcal vaccine (209/281; 74.3%), nonavailability of the vaccine in health facilities around respondents residence (42/281; 15.0%), religious concerns (8/281; 2.9%), cost of vaccination (12/281; 4.3%), universal concern about ineffectiveness of vaccination (6/281; 2.1%), and side effects (4/281; 1.4%). Further analysis showed that only maternal education (*P* = 0.00) and place of residence (*P* = 0.02) were significantly associated with correct knowledge of pneumonia aetiology, knowledge of dangers signs of pneumonia (*P* = 0.00), and uptake of vaccination (*P* = 0.01) against pneumonia (Table 2).

### 4.3. Knowledge and Perception of Danger Signs of Pneumonia

Respondents were asked to list all features that in their opinion signify danger when a child is suspected of having pneumonia. Fever 226 (20.6%), fast breathing 181 (16.5%), continuous cough 180 (16.4%), chest pain 67 (6.1%), and difficulty in breathing 66 (6.0%) were the most listed features. Also listed were cold body (i.e., hypothermia) 63 (5.7%), catarrh/running nose 43 (3.9%), weakness 41 (3.7%), chest wall indrawing 37 (3.4%), convulsion 33 (3.0%), and others 161 (14.7%). The others included but not limited to poor suck 33, excessive cry 28, abdominal distension 27, restlessness 18, stridor, that is, noisy breathing 15, unconsciousness 11, vomiting 10, watery stooling 9, dullness of face and body 6, and pale skin 5 (Table 3). Information source on perceived danger signs of respondents followed almost a similar pattern as causes of pneumonia described above except that previous experience of danger signs accounted for close to half, 224 (48.1%) of information source here. Others include electronic media in 99 (21.2%), Internet in 11 (2.4%), social interaction in 10 (2.1%), medical counseling in 86 (18.5%), and others in 35 (7.2%) respondents. Two hundred and thirty-two (56.3%) of respondents have experienced their perceived danger sign of pneumonia in one or more of their children. Fever 123 (24.5%), continuous cough 98 (19.5%), fast breathing 75 (14.9%), cold 34 (6.8%), convulsion 22 (4.4%), and difficulty breathing 17 (3.4%) top the list of the most experienced pneumonia danger signs among children of respondents (Table 3).

The WHO/UNICEF recognised danger signs most commonly known by respondents were fast breathing (60.5%) and difficulty in breathing (22.1%) while chest indrawing (12.4%) and stridor, that is, noisy breathing (5.0%), were less known to respondents (Table 3). Knowledge of at least one WHO/UNICEF recognised danger sign was seen in 304 (65.2%) of respondents while knowledge of 2, 3, and 4 recognised danger signs was seen in 219 (47.0%), 37 (7.9%), and 22 (4.7%) of respondents, respectively. One hundred and sixty-two (34.8%) had knowledge of none of the WHO/UNICEF danger signs. Just like cause and vaccine knowledge, maternal level of educational attainment (*P* = 0.04), and place of residence (*P* = 0.00) were significantly associated with knowledge of the WHO/UNICEF recognised pneumonia danger signs in children among respondents (Table 2).

### 4.4. Health Seeking Behaviour of Respondents for Suspected Pneumonia in Children

Respondent's first line of action when child developed suspected pneumonia was sought. Sixty-four percent of the 253 that responded to the question presented to the hospital, 78 (30.8%) either bought drugs over the counter or visited the patent medicine dealer for treatment, 10 (4.0%) consulted the traditionalist, and 3 (1.2%) took the child to the church for prayers and spiritual healing. First-line treatment option in all respondents ranged from antibiotics 52 (20.2%), cough syrups 119 (46.3%), vitamin C 7 (0.03%), drug combinations 46 (17.9%) [i.e., antibiotics + cough syrup, antibiotics + cough syrup + vitamin C, etc.], herbal concoction 9 (0.04%), and hospital admission 24 (9.4%). Majority of the children 202 (81.1%) recovered fully, 38 (15.3%) did not survive the experience, and 9 (3.6%) recovered but with complications. Caregivers with tertiary education (70.0%) used hospitals more compared to those with secondary (60%), primary (68%), and no education (60%) (*P* = 0.001). Conversely, caregivers with no formal education (14.0%) and those with primary education (2.0%) used traditional and/or spiritual treatment more compared to those with tertiary (0.0%) and secondary school education (0.0%) (*P* = 0.001). Finally, caregivers resident in rural area compared to those resident in semiurban areas sought care in a healthcare facility more (65.0% versus 59.0%), self-medicated less (28.0% versus 41.0%), and consulted traditionalist and/or spiritualist more (7.0% versus 0.0%) (*P* = 0.04) (Table 2).

## 5. Discussion

This study investigated the knowledge of caregivers and caregivers in Enugu about the aetiology and danger signs of pneumonia. It also sought to determine factors that influenced the knowledge and health seeking behaviour of caregivers for their under-5 children with probable pneumonia. The study showed high awareness (95%) and knowledge of potential fatality of pneumonia disease (97.7%) among respondents but poor knowledge of the aetiology (4.1%) and danger signs of probable pneumonia. A similar study in Thailand also showed inadequate knowledge of danger signs (7%) and causes (21%) of pneumonia among respondents [13]. The relatively poorer knowledge of causes of pneumonia in this study may be related to the higher number of caregivers without any formal education (29.2%) compared to 4.3% among respondents in the Thailand study. Another study in Uganda corroborated the poor knowledge among caregivers. It was noted in that study that none of the caregivers surveyed was able to mention the four standard danger signs of childhood pneumonia and only 9.4% of the respondents knew that lower chest wall indrawing was a danger sign in childhood pneumonia [14].

It was also shown in this study that older caregivers, caregivers with higher educational attainment, and those that are resident in semiurban areas had better knowledge of the cause and danger signs of pneumonia. This is hardly surprising as caregivers with these sociodemographic variables are more likely to have more experiences of childhood illnesses and/or are better informed about pneumonia. A hospital based study in Lagos University Teaching Hospital affirmed this finding [15]. It showed that caregivers with lower than tertiary education were more likely to have less knowledge on aspects of pneumonia disease. The Lagos study in addition showed that caregivers whose information source about pneumonia was from health personnel were more likely to have correct information on the cause of pneumonia compared to those from other sources. This fact was supported by this present study which revealed that respondents who obtained their information from medical personnel were more likely to have correct knowledge of aetiology and prevention of pneumonia compared to respondents that got their information from other sources.

Inquiries into the preventive strategies showed that majority of the respondents believed that adequate clothing and avoidance of cold food, drink, or environment prevents pneumonia disease in a child. This belief which is a common misconception among many lay people in Nigeria was also corroborated by the study in Lagos which found that 75.6% of mothers surveyed believed that cold was the main cause of pneumonia [15]. Furthermore, almost half of respondents in the present survey had knowledge of pneumonia vaccine with only 39.9% of these respondents having vaccinated their child against pneumonia. Similarly, only about a tenth of caregivers in our survey listed vaccination as a preventive strategy for pneumonia. This low uptake rate is expected as pneumococcal vaccines, which are relatively new additions to the national immunization schedules, are not widely available in public health facilities. Furthermore, even when available, they are not free like other routine vaccines in Nigeria.

Higher maternal educational attainment and residence in semiurban areas were significantly associated with knowledge about pneumococcal vaccine and uptake rate among children of surveyed caregivers. A study conducted in the Africa Center Demographic Site in South Africa showed that caregivers with secondary and tertiary education were 1.10 and 1.09 times, respectively, more likely to uptake Pneumococcal Conjugate Vaccine (PCV) for their children compared to caregivers with no education. It further showed that those caregivers resident in rural and periurban areas were 0.90 and 0.96 times less likely to uptake pneumococcal vaccine for their child. The author argued that distance from vaccination centers may have accounted for these findings [16].

Though majority of the respondents in our study took their children to hospital on suspicion of pneumonia, nearly 40% either self-medicated and/or consulted traditionalist or spiritualist as the first line of action. The finding that caregivers with lower education were more likely to engage in this behaviour is easily rationalized. Because these caregivers are more likely to be less informed and easily persuaded, they will more likely be influenced by advice from friends and relatives to seek nonmedical solutions for their child's illness (especially in the face of poverty). This is a fairly common practice in our society. It was further noted that caregivers in rural area were more likely to visit hospital, less likely to self-medicate, and also more likely to visit traditionalist and/or spiritualist compared to caregivers in urban areas. It could be further rationalized that because of the easy accessibility of various category of drug stores and ease of getting drugs over the counter in urban areas caregivers tended to patronize these drug dealers and self-medicate their children during illnesses. Similarly, because of the traditional belief which is inherent in rural communities and poorer accessibility to healthcare facilities, it is not surprising that caregivers in rural areas may more likely seek traditional and/or spiritualist help for their child's illness. Contrary to this finding, a community based comparative study in Dera district of Oromia Regional state, Ethiopia, showed that more caregivers in urban area (75.0%) sought healthcare for acute respiratory tract infection in their children from healthcare institution compared to those in rural areas (34.4%). Although no explanation was proffered by the authors for this finding, one could speculate that the higher number of caregivers with formal education in the urban category (28.7%) compared to caregivers in the rural category (3.6%) may have contributed to the higher healthcare seeking behaviour among caregivers in the urban compared to those in the rural setting [17] unlike our study where there is no significant difference in educational attainment between caregivers' resident in urban and rural area.

Finally, we observed in our study that fast breathing and difficulty in breathing (labored breathing) were the most known and experienced WHO recognized danger signs among respondents. This may be related to the ease of noticing these symptoms in children compared to chest wall indrawing and stridor. The proportion of caregivers who had knowledge of these signs was more than what was observed in a similar study in Uganda [14]. On the other hand, fever, fast breathing, and cough were the most perceived and experienced danger sign among respondents in this study. Khalida et al. [18] in their own study in Mirpur Khas district of Pakistan similarly noted that fever and cough (65.2%), followed by fast breathing and chest wall indrawing (59.4%), were the most perceived symptoms of pneumonia by mothers in the district. As noted earlier, this may be related to the ease of picking up these symptoms in children.

## 6. Limitation

Selection bias would have resulted due to the point enrollment because prospective study participants were required to gather at a preagreed location for consenting and enrollment. Household sampling and enrollment which could have significantly reduced selection bias could not be done during selection of respondents in the community due to unorganized household locations and difficult terrains in some communities. Also, recall bias could have led to errors in data measurement, classification, and analysis since some caregivers were interviewed on events that took place in the past.

## 7. Conclusion

This study demonstrated poor knowledge of the aetiology, danger signs, and prevention of childhood pneumonia amongst caregivers. Health education targeted at caregivers will enhance dissemination of information to fill this knowledge deficit and hopefully reduce morbidity and mortality from childhood pneumonia. Therefore the authors intend to embark on workshops to sensitize medical personnel on the need to educate the caregivers on the danger signs and prevention of childhood pneumonia.

## Figures and Tables

**Table 1:** Descriptive summary of study respondents.

Variables	*N* *n* (%)
*Maternal age *(*years*)	*N* = 444
20–30	158 (35.6)
31–40	120 (27.0)
>40	166 (37.4)
*Maternal educational attainment*	*N* = 461
Tertiary	76 (16.5)
Secondary	107 (23.2)
Primary	142 (20.5)
None	136 (29.2)
*Ever heard of pneumonia*	*N* = 464
Yes	440 (94.8)
No	24 (5.2)
*Socioeconomic class*	*N* = 466
Upper	103 (22.1)
Middle	83 (17.8)
Lower	280 (60.1)
*Number of living children*	*N* = 426
None	14 (3.3)
1–4	246 (57.7)
>4	166 (39.0)
*Knowledge of WHO/UNICEF danger sign*	*N* = 466
None	162 (34.8)
At least 1	304 (65.2)
*Experiences of pneumonia danger signs in child*	*N* = 466
Yes	232 (49.8)
No	180 (38.6)
Do not know	54 (11.6)
*Age of child at experience of danger sign*	*N* = 211
<1 yrs	118 (55.9)
1-2 yrs	81 (38.4)
3–5 yrs	12 (5.7)

**Table 2:** Maternal sociodemographic parameters and association with pneumonia-related knowledge and care seeking behavior.

Variables	Causes of pneumonia				Knowledge ≥1 danger signs of pneumonia^†^1^^				Knowledge of pneumonia vaccine				Child vaccinated with PCV^†^2^^				Where care was sought for child with signs of pneumonia				
	Correct	Incorrect	*N*	*P*	Yes	No	*N*	*P*	Yes	No	*N*	*P*	Yes	No	*N*	*P*	Hosp^†^3^^	Self^*∗*^	Other	*N*	*P*
	*n* (%)	*n* (%)			*n* (%)	*n* (%)			*n* (%)	*n* (%)			*n* (%)	*n* (%)			*n* (%)	*n* (%)	*n* (%)		
*Age *(*years*)	14	408	422		291	153	444		212	95	307		181	120	301		157	74	12	243	
20–30	4 (3)	147 (97)	151	.04	96 (61)	62 (39)	158	.09	71 (62)	43 (38)	114	.10	58 (75)	79 (25)	137	.09	47 (65)	23 (32)	2 (3)	72	.73
31–40	8 (7)	109 (93)	117		76 (63)	44 (37)	120		64 (76)	20 (24)	84		57 (84)	11 (16)	68		49 (67)	21 (29)	3 (4)	73	
>40	2 (1)	152 (99)	154		119 (72)	47 (28)	166		77 (71)	32 (29)	109		66 (69)	30 (31)	96		61 (62)	30 (31)	7 (7)	98	

*Education *	18	421	439		301	160	461		213	102	315		181	69	250		159	77	13	249	
Tertiary	10 (13)	66 (87)	76	.00	42 (55)	34 (45)	76	.04	30 (65)	16 (35)	46	.00	23 (66)	12 (34)	35	.01	19 (70)	8 (30)	0 (0)	27	.00
Secondary	3 (3)	101 (97)	104		69 (64)	38 (36)	107		36 (51)	35 (49)	71		26 (61)	17 (39)	43		31 (60)	21 (40)	0 (0)	52	
Primary	4 (3)	133 (97)	137		105 (74)	37 (26)	142		83 (75)	28 (25)	111		76 (84)	14 (16)	90		62 (68)	28 (30)	2 (2)	92	
None	1 (1)	121 (99)	122		85 (62)	51 (38)	136		64 (74)	23 (26)	87		56 (68)	26 (32)	82		47 (60)	20 (26)	11 (14)	78	

*SEC* ^†^4^^	18	426	444		302	162	466		218	102	320		185	69	254		162	68	13	253	
Upper	11 (11)	92 (89)	103	.00	56 (56)	45 (44)	103	.09	37 (60)	25 (40)	62	.05	28 (64)	16 (36)	44	.19	26 (70)	11 (30)	0 (0)	37	.34
Middle	3 (4)	77 (96)	80		55 (66)	28 (34)	83		37 (61)	24 (39)	61		28 (68)	13 (32)	41		29 (63)	16 (35)	1 (2)	46	
Lower	4 (2)	257 (98)	261		191 (68)	89 (32)	280		144 (73)	53 (27)	197		129 (76)	40 (24)	169		107 (63)	51 (30)	12 (7)	170	

*Number of children*	16	392	408		276	150	426		202	92	294		167	63	230		152	70	12	234	
None	2 (15)	11 (85)	13	.07	6 (43)	8 (57)	14	.10	2 (50)	2 (50)	4	.27	1 (100)	0 (0)	1	.42	3 (75)	1 (25)	0 (0)	4	.76
0–4	10 (4)	225 (96)	235		155 (63)	91 (37)	246		109 (66)	57 (34)	166		86 (69)	38 (31)	124		82 (68)	33 (28)	5 (4)	120	
>4	4 (3)	156 (97)	160		115 (69)	51 (31)	166		91 (73)	33 (27)	124		80 (76)	25 (24)	105		67 (61)	36 (33)	7 (6)	110	

*Residence *	18	426	444		304	162	466		218	102	320		185	69	254		162	78	13	253	
Rural	6 (2)	293 (98)	317	.02	179 (56)	140 (44)	319	.00	122 (53)	109 (47)	231	.00	150 (77)	45 (23)	195	.01	127 (65)	54 (28)	13 (7)	194	.04
Semiurban	12 (8)	133 (92)	147		103 (70)	44 (30)	147		66 (74)	23 (26)	89		35 (59)	24 (41)	59		35 (59)	24 (41)	0 (0)	59	

^†^1^^ ^†^1^*X*^^^WHO|UNICEF recognized danger signs; ^†^2^^PCV: Pneumococcal Conjugate Vaccine; ^†^3^^Hosp: Private/Public Hospital; ^*∗*^self: self-medication; other: mainly traditional/spiritual medications; ^†^4^^SEC: socioeconomic class.

**Table 3:** Knowledge of pneumonia danger signs by respondents (multiple responses allowed).

Pneumonia danger signs	
*WHO/UNICEF recognized*	*N* = 299
Fast breathing	181 (60.5)
Difficulty in breathing	66 (22.1)
Chest wall indrawing	37 (12.4)
Stridor (noisy breathing)	15 (5.0)
*WHO/UNICEF experienced*	*N* = 110
Fast breathing	75 (68.2)
Difficulty in breathing	17 (15.5)
Chest wall indrawing	10 (9.1)
Stridor (noisy breathing)	8 (7.2)
*Respondent perceived*	*N* = 1098
Fever	226 (20.6)
Fast breathing	181 (16.5)
Cough (continuous)	180 (16.4)
Chest pain	67 (6.1)
Difficulty in breathing	66 (6.0)
Cold/shivering	63 (5.7)
Catarrh/running nose	43 (3.9)
Weakness	41 (3.7)
Chest wall indrawing	37 (3.4)
Convulsion	33 (3.0)
Others^†^1^^	161 (14.7)
*Respondent experienced*	*N* = 503
Fever	123 (24.5)
Cough (continuous)	98 (19.5)
Fast breathing	75 (14.9)
Cold/shivering	34 (6.8)
Convulsion	22 (4.4)
Difficulty in breathing	17 (3.4)
Catarrh/running nose	17 (3.4)
Inability to suck	17 (3.4)
Chest pain	16 (3.2)
Posttussive vomiting	11 (2.2)
Others^†^2^^	73 (14.5)

^†^1^^ ^†^1^*X*^^^Poor suck 33, excessive cry 28, abdominal distension 27, restlessness 18, noisy breathing 15, unconsciousness 11, vomiting 10, and so forth.

^†^2^^Excessive cry 12, weakness 11, abdominal distension 11, poor suck 11, chest wall indrawing 10, diarrhoea 9, noisy breathing 8, and so forth.
